# Inhibitory Effect of β-Carotene on *Helicobacter pylori*-Induced TRAF Expression and Hyper-Proliferation in Gastric Epithelial Cells

**DOI:** 10.3390/antiox8120637

**Published:** 2019-12-11

**Authors:** Yongchae Park, Hanbit Lee, Joo Weon Lim, Hyeyoung Kim

**Affiliations:** Department of Food and Nutrition, Brain Korea 21 PLUS Project, College of Human Ecology, Yonsei University, Seoul 03722, Korea; pych3130@hanmail.net (Y.P.); beachmn@naver.com (H.L.); jwlim11@yonsei.ac.kr (J.W.L.)

**Keywords:** β-carotene, *Helicobacter pylori*, hyper-proliferation, NF-κB, tumor necrosis factor receptor-associated factor

## Abstract

*Helicobacter pylori* infection causes the hyper-proliferation of gastric epithelial cells that leads to the development of gastric cancer. Overexpression of tumor necrosis factor receptor associated factor (TRAF) is shown in gastric cancer cells. The dietary antioxidant β-carotene has been shown to counter hyper-proliferation in *H. pylori*-infected gastric epithelial cells. The present study was carried out to examine the β-carotene mechanism of action. We first showed that *H. pylori* infection decreases cellular IκBα levels while increasing cell viability, NADPH oxidase activity, reactive oxygen species production, nuclear factor kappa-light-chain-enhancer of activated B cells (NF-κB) activation, and TRAF1 and TRAF2 gene expression, as well as protein–protein interaction in gastric epithelial AGS cells. We then demonstrated that pretreatment of cells with β-carotene significantly attenuates these effects. Our findings support the proposal that β-carotene has anti-cancer activity by reducing NADPH oxidase-mediated production of ROS, NF-κB activation and NF-κB-regulated TRAF1 and TRAF2 gene expression, and hyper-proliferation in AGS cells. We suggest that the consumption of β-carotene-enriched foods could decrease the incidence of *H. pylori*-associated gastric disorders.

## 1. Introduction

The gram-negative bacillus *Helicobacter pylori (H. pylori*) is a human-specific pathogen that colonizes the mucus layer of the human stomach [1]. *H. pylori* chronically infects about one half of the world’s population and is the only bacterial species to have been classified as a class 1 carcinogen by the World Health Organization [2]. *H. pylori* infection causes hyper-proliferation of gastric epithelial cells, thus leading to the development of gastric cancer [3]. Determination of the pathway(s) by which *H. pylori* infection promotes cell proliferation and survival might lead to the development of therapeutics for prevention of gastric cancer. 

Our work has focused on the mechanism(s) by which dietary antioxidants inhibit *H. pylori*-induced oxidative stress, expression of inflammatory mediators and oncogenes, and cell proliferation in gastric epithelial cells [4,5,6,7,8,9,10,11]. In the present study, we investigated the potential role of specific members of the tumor necrosis factor receptor-associated factor (TRAF) family in *H. pylori*-induced cell proliferation. Previous studies have indicated that the expression of TRAF1 and TRAF2 is up-regulated in gastric cancer cells [12,13,14]. In particular, TRAF1 and TRAF2 form heterodimers [15] and mediate the activation of the transcriptional factor NF-κB, which in turn regulates TRAF1 and TRAF2 gene expression [16], as well as genes that play important roles in anti-apoptotic and inflammatory signaling pathways. Nuclear factor kappa-light-chain-enhancer of activated B cells (NF-κB) activation involves its release from its complex with cytoplasmic IκBα and translocation to the nucleus, a process that is triggered by reactive oxygen species (ROS) and involves ubiquitin-mediated IκBα degradation [17].

Previous studies have implicated nicotinamide adenine dinucleotide phosphate (NADPH) oxidase, and specifically, the reactive oxygen species (ROS) generated by NADPH oxidase, as potential causative factors in *H. pylori*-induced gastric injury [18,19,20]. It has been shown that gastric epithelial cells infected with *H. pylori* contain higher levels of NADPH oxidase activity and consequently, higher levels of ROS, leading to the degradation of IκBα and activation of NF-κB [4,21]. 

The antioxidant β-carotene—which is responsible for the orange color of many fruits and vegetables, such as carrots and sweet potatoes—inhibits cell growth and also induces apoptosis and cell cycle arrest in various cancers, such as breast cancer and colon cancer [22,23]. β-Carotene is known to reduce ROS levels in *H. pylori*-infected gastric epithelial cells [5]. Therefore, we hypothesized that β-carotene might inhibit *H. pylori*-induced up-regulation of TRAF1 and TRAF2 and thereby inhibit NF-κB activation in gastric epithelial cells. 

## 2. Materials and Methods 

### 2.1. Cell Line and Culture Conditions 

Gastric epithelial AGS cells (gastric adenocarcinoma, ATCC CRL 1739, Rockvile, MD, USA) were purchased from the American Type Culture Collection (Rockvile, MD, USA). The cells were grown in complete medium, consisting of RPMI 1640 medium (GIBCO, grand Island, NY, USA), supplemented with 10% fetal bovine serum (GIBCO), 2 mM glutamine, 100 U/mL penicillin, and 100 μg/mL streptomycin (Sigma-Aldrich, St. Louis, MO, USA). The cells were cultured at 37 °C under a humidified atmosphere of 95% air and 5% CO_2_.

### 2.2. Cell Culture with H. pylori Infection 

The *H. pylori* NCTC 11637 used in this study was a cagA- and vacA-positive standard strain [24]. It was obtained from the American Type Culture Collection and inoculated on chocolate agar plates (Becton Dickinson Microbiology Systems, Cockeysville, MD, USA) in an anaerobic chamber (BBL Campy Pouch System, Becton Dickinson Microbiology Systems, Franklin Lakes, NJ, USA) at 37 °C under microaerophilic conditions.

AGS cells were seeded and cultured overnight to reach 80% confluency. Prior to *H. pylori* infection, the cells were washed with antibiotic-free culture medium. *H. pylori* cells were harvested from the chocolate agar plates, suspended in antibiotic-free RPMI 1640 medium supplemented with 10% fetal bovine serum, and then added to the AGS cells. 

### 2.3. Plasmid Construction and Transfection 

The vector for expression of the dominant negative mutant TRAF1 gene (Δ139-416) was constructed by carrying out PCR amplification of the targeted TRAF1 coding sequence, digestion of the PCR product with KpnI/XhoI (Promega, Madison, WI, USA), followed by ligation of the resulting fragment with KpnI/XhoI-digested pcDNA3 plasmid (Invitrogen, Carlsbad, CA, USA). The oligonucleotides used in the PCR amplification for introduction of the KpnI and XhoI cleavage sites were GGTACCATGGCCCTGGAGCA and CTCGAGTTGGAGCTCCCTCAGG, respectively [25]. The cells were transfected with pcDNA, or with the pcDNA-containing dominant negative mutant TRAF1 by incubation with the FuGENE^®^ HD transfection reagent (Promega, Madison, WI, USA) for 16 h. 

The plasmid containing the mutated IκBα gene was prepared according to published procedure [26]. The cells were transfected with pcDNA, or with the plasmid encoding the mutated IκBα gene by incubation with FuGENE^®^ HD transfection reagent for 16 h. 

### 2.4. Experimental Protocol

The impact of *H. pylori* infection of AGS cells on cell viability, TRAF1 and TRAF2 gene expression, and NADPH oxidase activity, and on the levels of ROS, IκBα, and NF-κB was determined for cells treated for 2 h with 0.5 μM and 1 μM β-carotene prior to infection at a 1:50 AGS cells-to-*H. pylori* ratio. β-Carotene was purchased from Sigma-Aldrich and dissolved in DMSO (Sigma-Aldrich, St. Louis, MO, USA). AGS cells were infected with *H. pylori* at the specified AGS cell-to-*H. pylori* ratio (at a 1:20 or 1:50 AGS cells to *H. pylori* ratio) and incubation period (24 h or 48 h) prior to execution of the assays described below. For annexin V/ propidium iodide (PI) staining, the cells were infected with *H. pylori* (at a 1:20 or 1:50 AGS cells-to-*H. pylori* ratio) for 48 h. Control experiments were carried out with uninfected AGS cells (“None”) and with infected AGS cells treated with a vehicle for β-carotene (<0.1% DMSO) alone (“Control”). 

### 2.5. Determination of Cell Viability

The AGS cell viability was determined by using the trypan blue exclusion assay (0.2% trypan blue) to determine the cell count, and the MTT assay (thiazolyl blue; Sigma-Aldrich, St. Louis, MO, USA) to determine the percentage of viable cells. Cells were seeded at 1 × 10^4^ cell/mL in a 24-well culture plate and incubated overnight before adding MTT in phosphate-buffered saline (PBS). The cells were lysed by mixing them for 20 min with 2-propanol in 0.1% HCl using a shaker. Absorbance determinations were carried out with a microplate reader (Molecular Devices, Sunnyvale, CA, USA). 

### 2.6. Annexin V/Propidium Iodide (PI)-Staining Assay

Apoptosis was measured by flow cytometry using Annexin V/PI staining (BD Biosciences, San Jose, CA, USA). Cells were infected with *H. pylori* for 48 h. The cells were collected, washed with ice-cold PBS, and resuspended in 200 μL 1X binding buffer-containing Annexin V (1:50 according to the manufacturer’s instructions) and 20 ng/sample PI for 15 min at 37 °C in the dark. Then, the number of viable, apoptotic, and necrotic cells was quantified by flow cytometry (Becton Dickinson, Franklin Lakes, NJ, USA) and analyzed by the CellQuest software. Cells were excited at 488 nm, and the emissions of Annexin V at 525 nm and PI were collected through 610 nm band-pass filters. At least 10,000 cells were analyzed for each sample. Apoptosis rate (%) = (number of apoptotic cells) / (number of total cells observed) × 100.

### 2.7. Determination of TRAF1 and TRAF2 mRNA Using Real-Time PCR Analysis

The total cellular RNA was first isolated by using TRI reagent (Molecular Research Center, Inc., Cincinnati, OH, USA), and then converted to cDNA using a random hexamer (Promega, Madison, WI, USA), MuLV reverse transcriptase (Promega, Madison, WI, USA), and a heating protocol consisting of 23 °C for 10 min, 37 °C for 60 min, and 95 °C for 5 min. The cDNA was used for real-time PCR with specific primers for TRAF1, TRAF2 and GAPDH mRNA. The primers specific for TRAF1 sequence (GenBank accession number NM_005658.5) are 5’-CATGAGAGGGGAGTATGATG-3’ (forward primer) and 5’-GAAGAAGAGTGGGCATCCAC-3’ (reverse primer). The primers specific for TRAF2 sequence (GenBank accession number NM_021138.4) are 5’-TTCCCCTTAACTTGTGACGGC-3’ (forward primer) and 5’-CAATCTTGTCTTGGTCCAGCC-3’ (reverse primer). In addition, the primers specific for GAPDH sequence (GenBank accession number NM_001357943.2) are 5’-GAAGGTGAAGGTCGGAGTC-3’ (forward primer) and 5’- GAAGATGGTGATGGGATTTC-3’ (reverse primer). For PCR amplification, the cDNA was amplified by 45 denaturation cycles consisting of 95 °C for 30 s, annealing at 55 °C for 30 s, and extension at 72 °C for 60 s. During the first cycle, the 95 °C step was extended to 3 min. The GAPDH gene was amplified in the same reaction to serve as the reference gene. 

### 2.8. Preparation of Whole-Cell Extracts, Membrane Extracts, and Nuclear Extracts 

The cells were first trypsinized and then pelleted by centrifugation at 5000× g for 5 min. The pellets were suspended in lysis buffer (10 mM Tris (pH 7.4), 15 mM NaCl, 1% Nonidet P-40 (Sigma-Aldrich, St. Louis, MO, USA) and protease inhibitor complex (Complete; Roche, Mannheim, Germany) and extracted by drawing the suspension through a 1 mL syringe with several rapid strokes. The resulting mixtures were placed on ice for 30 min and then centrifuged at 13,000× *g* for 15 min. The supernatants were used as whole-cell extracts. To prepare membrane extracts, the supernatants were further centrifuged at 100,000× *g* for 1 h at 4 °C. The pellets were resuspended in lysis buffer (50 mM HEPES, 150 mM NaCl, 1 mM EDTA, and 10% glycerol (all from Sigma-Aldrich, St. Louis, MO, USA) and used as the membrane extracts. For the preparation of nuclear extracts, the cells were lysed in hypotonic buffer (10 mM HEPES, 1.5 mM MgCl_2_, 10 mM KCl, 1 mM DTT, 0.5 mM PMSF, 0.05% Nonidet P-40, and 0.1 mM EDTA), followed by centrifugation at 13,000× *g* for 10 min. The pellets were resuspended in nuclear extraction buffer (20 mM HEPES (pH 7.9), 420 mM NaCl, 0.1 mM EDTA, 1.5 mM MgCl_2_, 25% glycerol, 1 mM DTT, and 0.5 mM PMSF) on ice and then centrifuged. The supernatants were collected and used for NF-κB DNA-binding activity determinations. Protein concentrations were measured by using the Bradford assay (Bio-Rad Laboratories, Hercules, CA, USA).

### 2.9. Western Blot Analysis for TRAF1, TRAF2, and IκBα

Aliquots from whole-cell extracts were loaded onto 7–14% SDS polyacrylamide gels (30–40 μg protein/lane) and separated by electrophoresis under reducing conditions. The proteins were transferred onto nitrocellulose membranes (Amersham, Inc., Arlington Heights, IL, USA) by electroblotting. The transfer of protein was verified using reversible staining with Ponceau S (Sigma-Aldrich, St. Louis, MO, USA). The membranes were blocked using 3% non-fat dry milk in TBS-T (Tris-buffered saline and 0.2% Tween 20) and then incubated overnight at 4 °C with antibodies for TRAF1 (sc-1831, Santa Cruz Biotechnology, Dallas, TX, USA), TRAF2 (sc-876, Santa Cruz Biotechnology, Dallas, TX, USA), IκBα (sc-371, Santa Cruz Biotechnology, Dallas, TX, USA), and actin (sc-1615, Santa Cruz Biotechnology, Dallas, TX, USA) in TBS-T solution containing 3% dry milk. After washing the membranes with TBS-T, the primary antibodies were detected using horseradish peroxidase-conjugated secondary antibodies (anti-mouse, anti-rabbit, anti-goat), and visualized by exposure to BioMax MR film (Kodak, Rochester, NY, USA) using the enhanced chemiluminescence detection system (Santa Cruz Biotechnology). Actin served as a loading control. 

### 2.10. Determination of ROS Levels

AGS cells (1.5 × 10^5^ cells/well) were seeded in a six-well culture plate and cultured overnight. The cells were treated with β-carotene and then incubated with *H. pylori* for a specified time period, after which the cells were incubated for 1 h with 10 µg/mL of dichlorofluorescein diacetate (DCF-DA; Sigma-Aldrich, St. Louis, MO, USA). Next, the cells were washed twice, harvested with phosphate-buffered saline (PBS), and loaded in 96-well black plates. Fluorescence measurements were made with a fluorometer (VICTOR 5 Wallac 1420 multi-label counter, Perkin Elmer life and Analytical Sciences, Boston, MA, USA) by using 485 nm for excitation and monitoring the emission at 535 nm.

### 2.11. Determination of NADPH Oxidase Activity

The NADPH oxidase activity was determined using assay solutions containing 2 mM KCN, 10 µM lucigenin (Enzo Life Sciences, Plymouth Meeting, PA, USA), and 100 µM NADPH (Sigma-Aldrich, St. Louis, MO, USA) in 50 mM Tris-MES buffer (pH 7.0). The reaction was initiated by the addition of AGS cell membrane extracts containing 10 µg of protein. The proton emission was monitored for 5 min at 15 s intervals with a micrometerplate luminometer (Micro-Lumat LB 96 V luminometer, Berthold, NH, USA). In parallel, negative control measurements were carried out wherein the NADPH oxidase activities of the cytosolic extracts were measured. 

### 2.12. Electrophoretic Mobility Shift Assay (EMSA) for NF-κB 

The NF-κB gel shift oligonucleotide (5’-ACTTGAGGGGACTTTCCCAGGGC-3’) (Promega) was radiolabeled using [^32^P]-dATP (Amersham Biosciences, Piscataway, NJ, USA) and T4 polynucleotide kinase (GIBCO, Grand Island, NY, USA). The radiolabeled oligonucleotide was separated from unconsumed [^32^P]-dATP using a Bio-Rad purification column (Bio-Rad Laboratories, Hercules, CA, USA) eluted with Tris-EDTA buffer (pH 7.5). AGS cell nuclear extracts were incubated at 25 °C for 30 min with the [^32^P]-labeled oligonucleotide in buffer containing 12% glycerol, 12 mM HEPES (pH 7.9), 1 mM EDTA, 1 mM DTT, 25 mM KCl, 5 mM MgCl_2_, and 0.04 μg/mL poly[d(I-C)] (Sigma-Aldrich, St. Louis, MO, USA). The samples were subjected to electrophoretic separation at 4 °C on a nondenaturing, 5% acrylamide gel. The gel was dried at 80 °C for 2 h after which it was exposed at –80 °C to a radiography film enhanced by an intensifying screen.

### 2.13. Immunoprecipitation of the TRAF1-TRAF2 Complex

AGS cells were transfected with pcDNA, or with a plasmid encoding a dominant negative TRAF1 mutant gene, for 16 h and then infected with *H. pylori.* Following incubation for a specified period, the cells were lysed in 500 mM immunoprecipitation buffer (10 mM Tris-HCl (pH 7.4), 100 mM NaCl, 1 mM EDTA, 1 mM EGTA, Complete (Roche), 0.5% NP-40, 0.5% sodium deoxycholate (Sigma-Aldrich), and 10% glycerol). The lysate was centrifuged at 15,000× *g* for 15 min. Polyclonal antibody and protein G-agarose were added to the supernatant, and the mixture was incubated overnight at 4 °C. The protein G-antibody-antigen complex was collected, washed four times with immunoprecipitation buffer (150 mM NaCl, 10 mM Tris-HCl (pH 7.4), 1 mM EDTA, 1 mM EGTA, 0.5% NP-40 and 0.5% sodium deoxycholate), suspended in 50 µL of SDS sample-loading buffer and boiled for 10 min. The resulting mixture was chromatographed on a SDS-PAGE gel for Western blot analysis.

### 2.14. Statistical Analysis

All data are reported as the mean ± SE of four independent experiments. Statistical analysis was performed using SPSS 24.0 (SPSS, Chicago, IL, USA). One-way ANOVA, followed by Newman–Keul’s post hoc tests, were used for statistical analysis. A *p*-value of 0.05 or less was considered statistically significant.

## 3. Results

### 3.1. H. pylori Induces Expression of TRAF1 and TRAF2, IκBα Degradation, NF-κB Activation, and Cell Proliferation, but Does Not Induce Apoptosis in AGS Cells

**Figure 1:**
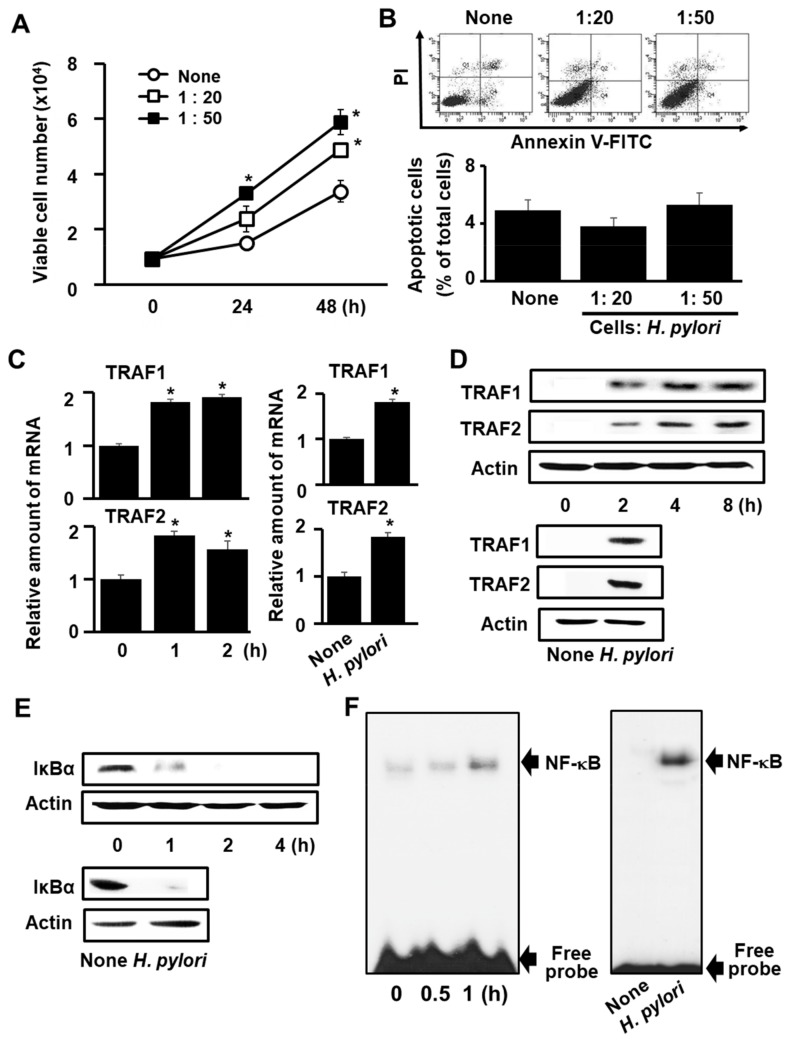
*H. pylori* induces the expression of TRAF1 and TRAF2, IκBα degradation, NF-κB activation, and hyper-proliferation, but does not induce apoptosis in AGS cells. (**A**) The number of viable AGS cells measured using the trypan blue exclusion test at 0 h, 24 h, and 48 h following infection at a 1:20 and a 1:50 AGS cells-to-*H. pylori* ratio. *p < 0.05 vs. none (**B**) Flow cytometric analysis results of *H. pylori*-induced apoptosis in AGS cells following infection by *H. pylori* in a 1:20 and a 1:50 AGS cells to *H. pylori* ratio for 48 h (upper panel). A statistical graph of annexin V/PI staining is shown (lower panel). Apoptotic cells include the Annexin V+/PI− cells and the Annexin V+/PI+ cells. (**C**) The relative amounts of TRAF1 and TRAF2 mRNA in AGS cells measured by real time PCR at 0 h, 1 h, and 2 h following infection by *H. pylori* in a 1:50 AGS cells to *H. pylori* ratio (left panel). At 1 h-culture, mRNA expression of TRAF1 and TRAF2 was determined in uninfected cells (None”) or in *H. pylori*-infected cells in a 1:50 AGS cells to *H. pylori* ratio (“*H. pylori*”) (right panel). The mRNA levels were normalized to that of GAPDH. * *p* < 0.05 vs. 0 h or none. (**D**) Western blot analysis of the levels of TRAF1 and TRAF2 proteins in AGS cells following infection by *H. pylori* in a 1:50 ratio and for 2 h, 4 h and 8 h periods (upper panel). At 4 h of culture, protein expression of TRAF1 and TRAF2 was determined in uninfected cells (None”) or in *H. pylori*-infected cells in a 1:50 AGS cells to *H. pylori* ratio (“*H. pylori*”) (lower panel). Actin was used as a loading control. (**E**) Western blot analysis of the levels of IκBα at 0 h, 1, 2, and 4 h of AGS cells incubation with *H. pylori* in a 1:50 AGS cells to *H. pylori* ratio (upper panel). At 1 h-culture, the levels of IκBα was determined in uninfected cells (None”) or in *H. pylori*-infected cells in a 1:50 AGS cells to *H. pylori* ratio (“*H. pylori*”) (lower panel). Actin was used as a loading control. (**F**) EMSA analysis was used for determination of the amount of active nuclear NF-κB in AGS cells following infection by *H. pylori* in a 1:50 ratio and for 0.5 h and 1 h periods (left panel). At 1 h-culture, active nuclear NF-κB levels were determined in uninfected cells (None”) or in *H. pylori*-infected cells in a 1:50 AGS cells to *H. pylori* ratio (“*H. pylori*”) (right panel). “Free probe” corresponds to the [^32^P]-oligonucleotide probe used in the shift assay.

To determine the experimental conditions best suited for the investigation of the effect of β-carotene on *H. pylori*-induced proliferation of AGS cells, cell viability was measured as a function of the *H. pylori* titer (ratio of AGS cells to *H. pylori* at 1:20 vs. 1:50) and the length of the infection period (24 h and 48 h). The results, reported in Figure 1A, reveal that the *H. pylori*-induced increase in the number of viable AGS cells is significantly greater at the AGS-to-*H. pylori* ratio of 1:50 than at the ratio of 1:20, independent of the length of the infection period. For an assessment of apoptosis induced by *H. pylori* infection, we examined the exposure of phosphatidylserine on the cell surface by using Annexin V/PI double-staining. Flow cytometry analysis revealed that *H. pylori* infection did not induce apoptosis at 48 h of culture, determined by the percentage of Annexin V-positive apoptotic cells/total cells (Figure 1B). Thus, further studies were performed at the AGS cells-to-*H. pylori* ratio of 1:50 for the incubation period optimal for each experiment. 

To determine whether *H. pylori* infection alters TRAF1 and TRAF2 gene expression, the amounts of TRAF1 and TRAF1 mRNAs were measured by real-time PCR analysis (Figure 1C), and the amounts of TRAF1 and TRAF2 proteins were measured by western blot analysis (Figure 1D). The results, shown in Figure 1B, indicate that following 2 h of *H. pylori* infection, the TRAF1 and TRAF2 mRNA levels had increased significantly. Likewise, the results shown in Figure 1D reveal that the amounts of TRAF1 and TRAF2 proteins are significantly increased by *H. pylori* infection. 

Next, we examined the effect of AGS cell infection with *H. pylori* on the levels of IκBα degradation and NF-κB activation. The results obtained from western blot analysis show that the level of IκBα (Figure 1E) was greatly reduced at 1 h following infection, and that at 2 and 4 h following infection, the amount of IκBα was reduced to below the detection limit. Consistent with this finding, the level of active nuclear NF-κB, measured using the electrophoretic mobility shift assay (EMSA), is significantly increased in AGS cells within 1 h of *H. pylori* infection (Figure 1F). Taken together, these results indicate that *H. pylori* infection increases cell viability by decreasing IκBα levels and thereby activating NF-κB, which in turn up-regulates TFA1 and TRAF2 gene expression. 

### 3.2. β-Carotene Inhibits H. pylori-Induced Expression of TRAF1 and TRAF2 and Hyper-Proliferation in AGS Cells

**Figure 2:**
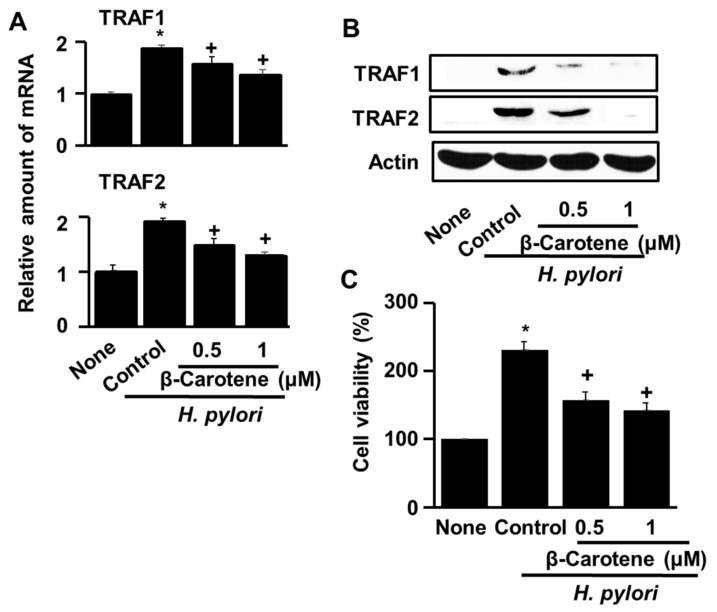
β-Carotene inhibits *H. pylori*-induced TRAF1 and TRAF2 gene expression and hyper-proliferation in AGS cells. The cells were pretreated with the indicated concentrations of β-carotene for 2 h, and then infected with *H. pylori* at a titer ratio of 1:50. The column “None” corresponds to uninfected, untreated cells, the column “Control” corresponds to untreated cells infected with *H. pylori* and the columns “0.5” and “1” correspond to cells incubated with 0.5 and 1.0 μM β-carotene, respectively prior to infection with *H. pylori*. (**A**) The relative amounts of TRAF1 and TRAF2 mRNA in AGS cells measured by real time PCR without *H. pylori* infection or with *H. pylori* infection at 1 h culture (at the ratio of AGS cells with *H. pylori*, 1:50). The mRNA levels were normalized to GAPDH mRNA. * *p* < 0.05 vs. none, ^+^ *p* < 0.05 vs. control. (**B**) Western blot analysis of TRAF1 and TRAF2 in AGS cells without *H. pylori* infection, or with *H. pylori* infection in the absence and presence of β-carotene at 4 h of culture. Actin was used as a loading control. (**C**) The number of viable AGS cells measured without *H. pylori* infection or with *H. pylori* infection in the absence and presence of β-carotene at 48 h of culture. The cell viability was measured using the MTT assay. * *p* < 0.05 vs. none, ^+^ *p* < 0.05 vs. control.

To investigate the effect of β-carotene on the expression of TRAF1 and TRAF2 genes in *H. pylori*-infected AGS cells, the levels of TRAF1 and TRAF2 mRNAs and proteins were determined. Figure 2A show that following *H. pylori* infection for 1 h, the TRAF1 and TRAF2 mRNA levels were increased which reduced by β-carotene dose-dependently. Similarly, Figure 2B reveal that the amounts of TRAF1 and TRAF2 proteins in AGS cells infected for 4 h were decreased by β-carotene in a dose-dependent manner. 

To correlate the observed effect of β-carotene on *H. pylori*-induced up-regulation of the TRAF1 and TRAF2 genes (Figure 2A and Figure 2B) with the effect of β-carotene on *H. pylori*-induced hyper-proliferation in AGS cells, cell viability measurements were made. The results obtained, and shown in Figure 2C, reveal that β-carotene (0.5 and 1.0 μM) reduces the number of viable cells in *H. pylori*-infected AGS cells in a dose-dependent manner. Taken together, these results suggest that β-carotene inhibits *H. pylori*-induced cell proliferation by inhibiting the induced up-regulation of TRAF1 and TRAF2 gene expression in AGS cells. 

### 3.3. β-Carotene Inhibits H. pylori-Induced ROS Production, NADPH Oxidase Activation, IκBα Degradation, and NF-κB Activation in AGS Cells

**Figure 3:**
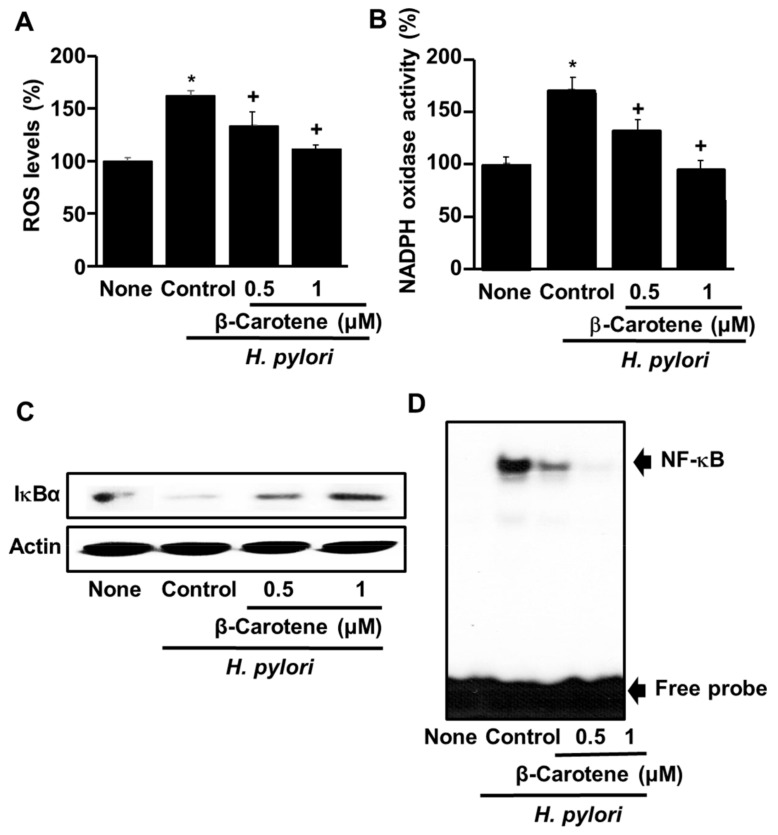
β-Carotene inhibits the effects of *H. pylori* infection of AGS cells on cellular ROS, NADPH oxidase, IκBα, and NF-κB. The cells were pretreated with the indicated concentrations of β-carotene for 2 h, and then infected with *H. pylori* at the titer ratio of 1:50 for 1 h. The column “None” corresponds to uninfected cells, the column “Control” corresponds to untreated cells infected with *H. pylori*, and the columns “0.5” and “1” correspond to cells incubated with 0.5 and 1 μM β-carotene, respectively, prior to infection with *H. pylori*. (**A**) ROS levels determined with the DCF assay. * *p* < 0.05 vs. none, ^+^ *p* < 0.05 vs. control. (**B**) NADPH oxidase activity determined with the Lucigenin assay. * p < 0.05 vs. none, ^+^ *p* < 0.05 vs. control. (**C**) IκBα levels determined by western blot analysis. Actin was used as the loading control. (**D**) NF-κB DNA-binding activity determined with the EMSA assay.

To determine the effect of β-carotene on *H. pylori*-induced ROS production in AGS cells, the levels of ROS were measured using the DCF-DA assay. Figure 3A show that β-carotene pre-treatment inhibits, in a dose-dependent manner (0.5 vs. 1 μM), the increase in ROS that occurs upon *H. pylori* infection. Next, the impact of β-carotene on the *H. pylori*-induced increase in NADPH oxidase activity was measured. As shown in Figure 3B, the NADPH oxidase activity in the infected AGS cells was significantly reduced by β-carotene in a dose-dependent manner. 

Lastly, we examined the effect of β-carotene on the levels of IκBα and active nuclear NF-κB. AGS cells infected by *H. pylori* were found to have a lower level of IκBα (Figure 3C) and higher level of nuclear NF-κB DNA-binding activity (Figure 3D) relative to uninfected cells. Treatment of the AGS cells with 0.5 or 1 μM β-carotene prior to infection significantly increased the level of IκBα (Figure 3C) in infected cells in a dose-dependent manner while decreasing the level of active nuclear NF-κB (Figure 3D). Taken together, these results indicate that β-carotene inhibits *H. pylori*-induced cell proliferation by inhibiting *H. pylori*-induced NADPH oxidase-mediated ROS production, and thus *H. pylori*-induced down-regulation of IκBα, and up-regulation NF-κB activation and TRAF1 and TRAF2 gene expression in AGS cells. 

### 3.4. Transfection of AGS Cells with a Mutated IκBα Gene Inhibits H. pylori-Induced Expression of TRAF1 and TRAF2 and NF-κB Activation 

**Figure 4:**
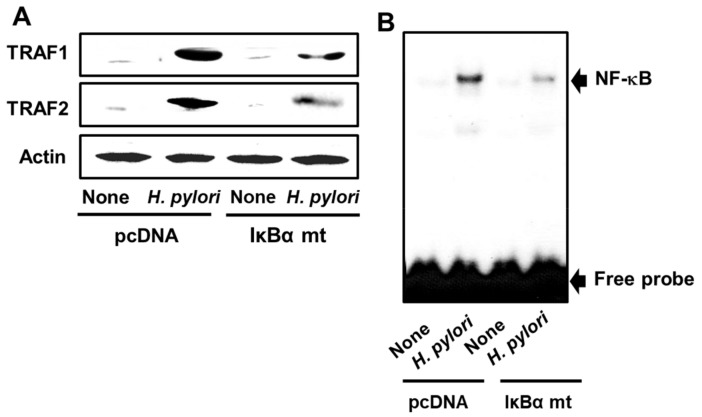
Transfection of AGS cells with an IκBα mutant gene inhibits *H. pylori*-induced expression of TRAF1 and TRAF2 and NF-κB activation. AGS cells that were transfected for 16 h with the control vector (“pcDNA”), or with the plasmid containing the IκBα mutant gene (“IκBα mt”), and then incubated with *H. pylori* at the titer ratio of 1:50 for 4 h (**A**), or 1 h (**B**). “*H. pylori*” corresponds to samples derived from infected cells, whereas “None” corresponds to samples derived from uninfected cells. (**A**) Western blot analysis of the levels of TRAF1 and TRAF2 with actin serving as the loading control. (**B**) The nuclear NF-κB DNA binding activity determined using the EMSA assay.

It has been previously shown that transfection of gastric epithelial cells with a mutated IκBα gene that encodes a dominant negative IκBα protein mutant inhibits NF-κB activation [25]. Here, we used the transfected IκBα gene mutant to block *H. pylori*-induced NF-κB activation in AGS cells. As shown by the results of the western blot analysis in Figure 4A, the levels of TRAF1 and TRAF2 are significantly lower in the IκBα gene mutant-transfected, *H. pylori*-infected AGS cells than in the native *H. pylori*-infected AGS cells. In addition, the level of nuclear NF-κB DNA binding activity was found to be lower in the *H. pylori*-infected AGS cells transfected with the IκBα gene mutant than in native *H. pylori*-infected AGS cells (Figure 4B). Taken together, these results correlate the *H. pylori*-induced activation of NF-κB with the up-regulation of TRAF1 and TRAF2. 

### 3.5. Transfection of Dominant Negative Mutant TRAF1 Gene Decreased Interaction of TRAF1 and TRAF2 and Cell Proliferation in H. Pylori-Infected AGS Cells 

**Figure 5:**
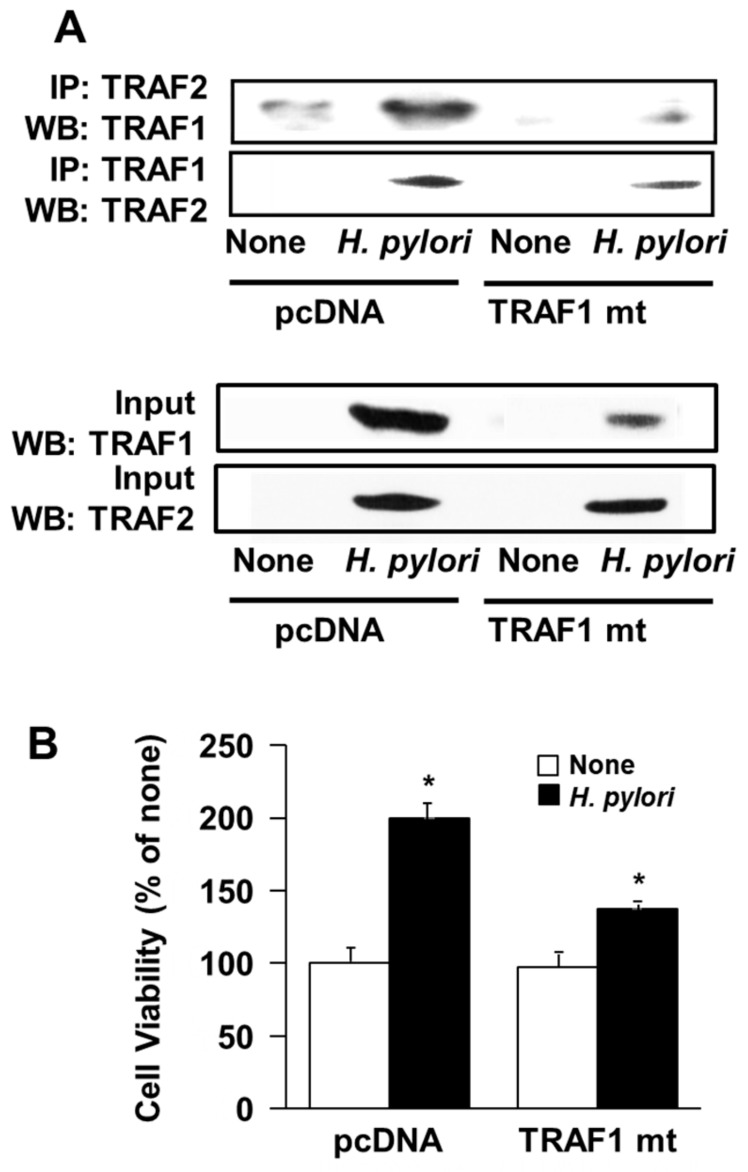
Transfection with the dominant negative mutant TRAF1 gene decreases the interaction of TRAF1 and TRAF2 and cell proliferation in *H. pylori*-infected AGS cells. The cells were transfected with control vector (“pcDNA”) or plasmid containing the dominant negative mutant TRAF1 gene (“TRAF1 mt”) for 16 h and then cultured with (“*H. pylori*”) or without (“None”) *H. pylori* at the titer ratio of 1:50 for 4 h (**A**) or 48 h (**B**). (**A**) Western blot analysis of the TRAF1- or TRAF2-antibody immunoprecipitate from whole-cell extracts of the cells transfected with control vector (“pcDNA”) or dominant negative mutant TRAF1 expression vector (“TRAF1 mt”), using TRAF1 and TRAF2 antibodies for visualization (upper panel). The cellular lysate (“Input”) served as the protein expression control (lower panel). (**B**) The percentage ratio of viable AGS cells transfected with control vector (“pcDNA”) or dominant negative mutant TRAF1 expression vector (“TRAF1 mt”) and in culture with vs. without *H. pylori*. The cell viability was measured using the MTT assay. * *p* < 0.05 vs. corresponding none.

To determine whether *H. pylori*-induced AGS cell proliferation required TRAF1 and TRAF2 association, the effect of overexpression of the dominant negative mutant TRAF1 gene was tested. The TRAF1 mutant lacks the N-terminal region that serves as the docking site for TRAF2 binding [20], and can thus interfere with TRAF1-TRAF2 complex formation. The results from the western blot analysis of the protein sample obtained by TRAF1 antibody- or TRAF2 antibody-induced immunoprecipitation of the TRAF1-TRAF2 complex are shown in Figure 5A. These results demonstrate that transfection of AGS cells with the dominant negative mutant TRAF1 gene decreases the interaction of TRAF1 with TRAF2. 

Next, the impact of transfection of dominant negative mutant TRAF1 gene on *H. pylori*-induced AGS cell proliferation was examined. The results reported in Figure 5B reveal that the dominant negative mutant TRAF1 reduces cell viability in cells infected with *H. pylori*. Taken together, the results indicate that the interaction between TRAF1 and TRAF2 is critical to the *H. pylori*-induced increase in AGS cell viability. 

## 4. Discussion

*H. pylori* infection induces hyper-proliferation of the human gastric epithelial cells [27]. The hyper-proliferation caused by *H. pylori* infection is an important feature of gastric cancer [28]. Previous studies suggest that a low density of *H. pylori* infection promotes AGS cell proliferation, whereas a high density of *H. pylori* infection induces AGS cell apoptosis [29]. In addition, *H. pylori* infection results in the generation of ROS, which is a key factor in *H. pylori*-induced gastric carcinogenesis [30]. 

Overexpression of TRAF1 is known to induce the development of gastric carcinogenesis [31,32]. In the present study, transfection of the dominant negative mutant TRAF1 gene inhibited *H. pylori*-induced hyper-proliferation in AGS cells. These results strongly suggest that overexpression of TRAF1 induced by *H. pylori* may be involved in the pathogenesis of gastric cancer. β-Carotene inhibited *H. pylori*-induced expression of TRAF1 and TRAF2. Therefore, our findings indicate that β-carotene may inhibit *H. pylori*-induced hyper-proliferation via down-regulation of TRAF1 and TRAF2. 

Previous studies have demonstrated that NF-κB regulates the transcription of TRAF1 and TRAF2 [33]. In the present study, transfection of a mutated IκB α gene inhibited *H. pylori*-induced expression of TRAF1 and TRAF2. These results provide evidence that NF-κB regulates the transcription of TRAF1 and TRAF2 genes in *H. pylori*-infected AGS cells. In addition, we showed that β-carotene inhibits activation of NF-κB in *H. pylori*-stimulated AGS cells. Therefore, β-carotene may inhibit *H. pylori*-induced expression of TRAF1 and TRAF2 by suppression of NF-κB activation. 

Several studies have shown that ROS regulates NF-κB activation via phosphorylation, ubiquitination, and subsequent proteasomal degradation of the inhibitory protein IκBα [34,35]. Consequently, this leads to the expression of several genes regulated by NF-κB, which promotes cell survival through inhibition of apoptotic pathways [36]. 

β-Carotene has been demonstrated to have anti-cancer therapeutic effects in various cells and tissues, and thus might serve as a therapeutic in the prevention or treatment of cancer [37]. Notably, β-carotene has been shown to inhibit cell growth and induce apoptosis in cancer cell lines [38,39]. In addition, β-carotene inhibited NF-kB activation in bone marrow-derived monocytes/-macrophages (BMM) [40], lipopolysaccharide (LPS)–stimulated macrophages [41], and hydrogen peroxide-exposed AGS cells [42]. Although the effects of β-carotene on the expression of TRAF1 or TRAF2 have not yet been reported, antioxidant nutrient γ-tocotrienol inhibited tumor necrosis factor alpha (TNF)-induced expression of NF-kB activation and expression of TRAF1 and TRAF2 in various human cells, such as myeloid KBM-5 cells, lung adenocarcinoma H1299 cells, embryonic kidney A293 cells, and breast cancer MCF-7 cells [43]. δ-Tocotrienol and quercetin suppressed NF-kB-regulated TRAF1 expression in LPS-stimulated macrophages [44]. Moreover, dietary antioxidants, such as phenolics extracted from tartary buckwheat bran and astaxanthin, inhibited TRAF2 expression through their antioxidant effects in human breast cancer MDA-MB-231 cells [45] and liver tissues of mice [46]. 

Our results show that β-carotene decreased *H. pylori*-induced expression of TRAF1 and TRAF2 by reducing ROS levels and NF-kB activation in AGS cells. Our finding suggests that β-carotene inhibits *H. pylori*-induced expression of TRAF1 and TRAF2 through its antioxidant activity. Given that TRAF1 and TRAF2 are involved in cell proliferation signaling, β-carotene inhibition of *H. pylori*-induced up-regulation of the TRAFs may contribute to the inhibitory effect of β-carotene on the hyper-proliferation caused by *H. pylori* infection in AGS cells.

In the present study, we used different time-points for determination of cell viability, NF-kB activation, and TRAF expression, since cell proliferation is the down-stream effect of NF-kB-regulated TRAF expression. Also, NF-kB activation is the upstream event of TRAF expression in *H. pylori*-infected cells. This hypothesis was supported by the results in Figure 1 showing that increased NF-kB DNA binding and decreased IkBα were evident at 1 h. TRAF mRNA expression was increased from 1 h, while protein expression of TRAF increased at 2 h, which continued until 8 h in *H. pylori*-stimulated cells. As shown in Figure 1A, at the ratio of AGS cells: bacterium, 1:50, viable cell number was significantly increased at 24 h and 48 h. Since cell proliferation is the late event by *H. pylori* infection, changing signaling molecules, such as NF-kB and TRAF gene expression, were monitored at an early stage, and cell proliferation was determined at later stages, such as at 24 h and 48 h. 

The high concentration of β-carotene alone (>2.5 μM) did not lead to decreased ROS and superoxide levels in macrophages, neutrophils, and endothelial cells [47,48,49]. Palozza et al. reported that 5 μM β-carotene did not change NF-κB DNA-binding activity in human leukemia and colon adenocarcinoma cells [50]. Based on these studies, treatment of 1 μM β-carotene alone, used in the present study, may not affect ROS levels and NF-κB activities in uninfected AGS cells. Further study should be performed to determine whether 1 μM β-carotene reduces ROS levels, NADPH oxidase activity, and NF-kB activity in uninfected AGS cells. 

The results from the present study show that β-carotene inhibits *H. pylori*-induced increases in cell viability, ROS production, NADPH oxidase activity, IκBα degradation, active nuclear NF-κB, and TRAF1 and TRAF2 gene expression, as well as protein–protein interaction in gastric epithelial AGS cells.

A meta-analysis study showed the dietary intake of β-carotene significantly reduced the risks of gastric cancer by 48% [51]. In a case control study in Korea, a higher intake of dietary β-carotene (4 mg/day) reduced risks of gastric cancer by 20% [52]. In a 7.2-year prospective cohort study in Sweden, dietary β-carotene intake (5 mg/day) reduced relative risks of gastric cancer by 45% [53]. Another case control study in Korea reported that a high intake of β-carotene (odds ratio (OR) = 0.54) exhibited highly significant inverse associations with gastric cancer among the *H. pylori*–infected subjects [54]. In addition, the mean serum levels of β-carotene were lower among *H. pylori*-positive individuals than *H. pylori*-negative individuals (decrease of 27.6%) [55]. From these studies, we can postulate that high dietary β-carotene intake (> 4 mg/day) may reduce *H. pylori*-associated infection and the risk of gastric cancer-associated *H. pylori* infection.

## 5. Conclusions

In summary, the present study examined the inhibitory mechanism of β-carotene on *H. pylori*-induced cell proliferation in relation to TRAF expression. In particular, this study provides evidence that β-carotene inhibits *H. pylori*-induced cell proliferation and expression of TRAF1 and TRAF2 by suppressing NADPH oxidase activity and NF-kB activation in gastric epithelial AGS cells. Conclusively, β-carotene inhibits NADPH oxidase-mediated ROS production and NF-kB activation, expression of TRAF1 and TRAF2, and hyper-proliferation in *H. pylori*-stimulated AGS cells. Based on these findings, we propose that dietary supplementation with β-carotene-rich foods may prevent or delay the development of gastric diseases, including gastric cancer, associated with *H. pylori* infection.
